# Role of FTO in Adipocyte Development and Function: Recent Insights

**DOI:** 10.1155/2015/521381

**Published:** 2015-12-16

**Authors:** Myrte Merkestein, Dyan Sellayah

**Affiliations:** ^1^Department of Physiology, Anatomy and Genetics, University of Oxford, Oxford, Oxfordshire OX1 3PT, UK; ^2^School of Biological Sciences, University of Reading, Reading, Berkshire RG6 6AS, UK

## Abstract

In 2007, *FTO* was identified as the first genome-wide association study (GWAS) gene associated with obesity in humans. Since then, various animal models have served to establish the mechanistic basis behind this association. Many earlier studies focussed on FTO's effects on food intake via central mechanisms. Emerging evidence, however, implicates adipose tissue development and function in the causal relationship between perturbations in FTO expression and obesity. The purpose of this mini review is to shed light on these new studies of FTO function in adipose tissue and present a clearer picture of its impact on obesity susceptibility.

## 1. Introduction

Obesity has risen to become the major health crisis of the current and potentially future generations. The latest figures from the World Health Organization (WHO) reveal a global estimate of 1.4 billion overweight individuals [1]. Most troubling is the devastating socioeconomic impact of obesity and related metabolic disturbances [2]. The WHO has estimated that 2–7% of global spending on health care is driven by a high body mass index (BMI) [3]. One such example is the financial strain being applied to the UK's National Health Service by obesity [4]. It is clear that more effective strategies are required, in terms of public policy, medical treatment, and biomedical research, to provide financially sustainable health care to be able to deal with the obesity crisis head-on. In recent years, genome-wide association studies (GWAS) have offered renewed promise in the quest to understand the genetics of obesity which has for too long eluded modern science.

In 2007, several independent GWAS and population based approaches identified associations between SNPs in intron 1 of* FTO* and human obesity in various European populations [5–8]. Since then, many studies have confirmed the association between SNPs in intron 1 of* FTO* and BMI in non-European populations, notable East Asians [9–11], South Asians [12–16], Africans [17], Hispanics [18, 19], and Native Americans [20], clearly demonstrating that the influence of FTO SNPs on obesity is a global trend. No association has been found between these obesity-associated SNPs and* FTO* expression levels in adipose tissue [21–25]. The fact that the obesity-associated* FTO* SNPs are intronic has led to the notion that the SNPs may affect obesity through influencing the expression of genes proximal to* FTO* in the locus, namely,* RPGRIP1L*,* IRX3*, and* IRX5* [26–29]. The obesity-associated allele of SNP rs8051036 has been suggested to decrease* RPGRIP1L* expression via reduced affinity for a transcriptional activator [26].* IRX3* expression has recently been documented to account for the obesity association with* FTO* SNPs in human cerebellar tissue [28] and in the pancreas in zebrafish [30]. Furthermore, long-range functional connections were observed between enhancers within the obesity-associated* Fto* interval and* Irx3* expression (and not* Fto*) in adult mouse brain tissues [28]. Another recent study found that an obesity-associated SNP in intron 1 of* FTO* was located in a long enhancer region in preadipocytes specifically. The risk allele disrupted the binding of the gene regulator* ARID5b*, which in turn leads to increased expression of* IRX3* and* IRX5* in preadipocytes [29].

These studies indicate that the obesity-associated region in intron 1 is likely to regulate the expression of various genes, which might be tissue- and developmental stage-specific. Future studies will address how the obesity-associated SNPs contribute to increased adiposity in other tissues and at early developmental stages. Thus, it remains a possibility that the obesity associated with the SNP is mediated by FTO during development or in peripheral tissues. As* FTO* was considered the “obesity-gene” in 2007 when the first GWAS papers were published, many research groups sought out to examine FTO's role in the regulation of body weight. Regardless of whether the obesity-associated SNP affects* FTO* expression levels, these studies have clearly proven an important role of FTO in the regulation of body weight, independent of* IRX3*,* IRX5*, and* RPGRIP1L*.

One important peripheral tissue that has increasingly been shown to be consequential to metabolic regulation and obesity susceptibility is adipose tissue. Interestingly, while it has been long established that* FTO* is highly expressed in adipose tissues [5], its function remained largely obscured. The purpose of this review is to examine the emerging role of FTO in adipose tissue and its relevance to obesity susceptibility.

## 2. Mouse Models of FTO Function

The first murine model of global germline* Fto* loss was described by Fischer et al. in 2009 [31].* Fto* deficient mice exhibited high perinatal lethality as well as postnatal growth retardation. Furthermore,* Fto* deficient mice had reduced lean and fat mass with respect to wild type mice. Intriguingly, fat mass was progressively reduced over the time in* Fto* deficient mice, to the extent that by 15 months of age* Fto* deficient mice were almost totally void of gonadal white adipose tissue (gWAT). Fischer and colleagues demonstrated that* Fto* deficiency causes relatively increased food intake as well as energy expenditure, potentially due to enhanced sympathetic tone resulting from elevated serum noradrenalin levels. The effect on energy expenditure in* Fto* deficient mice might have been due to the way in which the data were corrected to lean mass [32]. Others did not find an increase in energy expenditure in* Fto* deficient mice [33]. Interestingly, a model in which a point mutation in* Fto* resulted in reduced FTO protein expression and catalytic activity resulted in reduced lean mass and fat mass without any effects on perinatal lethality [34]. Paradoxically, adult onset* Fto* deficiency in mice (6 weeks of age) resulted in increased adiposity compared to wild type mice, with no effects on lean mass [33]. In line with these findings, a recent study reported increased body weight and adiposity of* Fto* knockout mice compared to wild type mice in response to a high fat diet [35].

To compound the inconsistency in body composition phenotypes with* Fto* deficiency, neural knockout of* Fto* led to a reduction in body weight accompanied by a decrease in lean mass, not fat mass [36], reflecting some of the germline FTO-KO models. However, adult onset knockout of* Fto* in the mediobasal hypothalamus decreased body weight without affecting body composition [33].

*Fto* overexpression models appear to be more consistent in describing an enhanced adiposity in* Fto* overexpression compared to wild type mice, an effect which is most pronounced on a high fat diet. Church and colleagues documented that* Fto* overexpression is accompanied by increased gWAT mass and increased adipocyte size [37], while Merkestein et al. revealed that adipocyte hypertrophy is preceded by increased gWAT hyperplasia in response to HFD, with respect to wild type mice demonstrating that FTO influences adipogenesis [38]. Conversely,* Fto* deficient mice have been shown to have reduced adipocyte size, both under chow conditions [31] and in response to high fat diet [39]; however, effects on adipose tissue hyperplasia were not studied in these models.

Although many questions remain to be answered and a clear understanding of the effects of FTO on body composition remains to be established, it is beyond doubt that FTO exerts an influence over adiposity and it is plausible that FTO directly regulates adipocyte development and metabolism.

## 3. Regulation of FTO Expression

The expression of* FTO* in adipose tissue is likely regulated in various ways. There is ample evidence for crosstalk between FTO and the LepRb-STAT3 signalling pathway and the involvement of the p110 isoform of the CUX1 transcription factor.

*FTO* risk alleles have been associated with changes in circulating levels of the appetite regulating hormones leptin and ghrelin. Several papers have shown that* FTO* risk allele is associated with increased circulating leptin levels [40–47]. However, this association appears to be mediated via increased adiposity, as in several studies the association disappears when correcting for BMI [42, 44–46]. Another study reported an association of an* FTO* risk allele with a decrease in circulating leptin levels which was independent of BMI in older participants [48]. Carriers of* FTO* risk alleles have been shown to have a reduced postprandial suppression of circulating acyl-ghrelin levels [49]. Similarly, another study found increased plasma ghrelin levels after overnight fast in people with* FTO* risk alleles [48].

Leptin increased* FTO* expression in cardiomyocytes, which was dependent on LepRb-STAT3 signalling and a subsequent increase in the p110 CUX1 isoform [50]. Overexpression of* FTO* in the arcuate nucleus of the hypothalamus in rats resulted in an increase in STAT3 mRNA [51]. On the other hand, leptin activated the STAT3 signalling pathway and reduced* FTO* expression in the arcuate nucleus, which was dependent on the LepRb receptor [52]. In hepatocytes, leptin administration, LepRb overexpression, and activation of the STAT3 pathway with IL6 induced FTO protein expression, whereas knockdown of STAT3 inhibited leptin-induced* FTO* mRNA expression. Conversely, overexpression of* FTO* reduced leptin-induced STAT3 phosphorylation and affected downstream events of this signalling pathway. Furthermore, overexpressing of* Fto in vivo* in mouse livers affected STAT3 phosphorylation and its downstream effects in a similar way [53].

Further evidence of the crosstalk between leptin and FTO comes from animal studies. In response to HFD,* Fto* deficient mice did not develop leptin resistance, whereas wild type mice did [35]. Furthermore, knocking out* Fto* improved the features of the metabolic syndrome normally observed in leptin deficient ob/ob mice [54].

Intron 1 of the* FTO* gene contains a binding site for the p110 isoform of the CUX1 transcription factor. The obesity-associated rs8050136 SNP is located in this region. Binding of the p110 CUX1 isoform enhances* FTO* expression [26]. Therefore, this transcription factor is likely to regulate* FTO* expression.

The* FTO* promoter region has also been shown to contain a C/EBP*α* binding site and C/EBP*α* promoted* FTO* expression in HEK293 and HeLa cells [55]. Furthermore, miR-33 was shown to regulate* FTO* expression. miR-33 is transcribed from an intronic region within* SREBF2*. SREBF2 is a transcriptional activator of many genes involved in the synthesis and uptake of cholesterol, triglycerides, fatty acids, and phospholipids. miR-33 is expressed in many tissues in the chicken, including adipose tissue. Knocking down miR-33 in primary chicken hepatocytes increased the expression of* FTO* [56].

## 4. FTO and Adipogenesis

Several studies have examined the expression of* FTO* during adipogenesis, the process by which new adipocytes are formed from preadipocytes, which in turn derive from mesenchymal stem cells. This process which has been well documented* in vitro* occurs in 7–10 days. In cultured preadipocytes and MEFs,* Fto* has been consistently shown to be highly expressed in the early phase of adipogenesis and to decline during the course of adipogenesis* in vitro* [24, 57–59].

These studies indicated a role for FTO in the adipogenic process. Indeed, knockdown of* Fto* decreased adipogenesis in 3T3-L1 preadipocytes [58, 60] and in porcine preadipocytes [61]. MEFs from* Fto*-KO mice exhibited reduced adipogenic potential, whereas overexpression of* Fto* led to an enhanced adipogenic program in primary murine preadipocytes, 3T3L1 preadipocytes, and porcine preadipocytes [38, 60, 61].

In line with these findings, gonadal WAT from* Fto* overexpressing mice fed a high fat diet for 8 weeks from weaning contained an increased number of adipocytes compared with WT mice; however, no differences in adipocyte number were evident between* Fto* overexpression mice and WT mice at weaning, clearly demonstrating that FTO promotes obesogenic adipogenesis in adulthood but plays no role in developmental adipogenesis [38]. This is important as obesogenic adipogenesis, which occurs in response to HFD in adulthood, has recently been shown to operate via distinct signalling mechanisms and requiring different subpopulations of preadipocytes [62]. Notably, the AKT2/PI3K signalling pathway has been evidenced to be essential for obesogenic adipogenesis but not required for developmental adipogenesis, possibly alluding to the insulin signalling aspect of obesogenic adipogenesis which is clearly dependent on dietary factors, in contrast to developmental adipogenesis which responds to developmental cues that predominate during organogenesis [62].

### 4.1. Catalytic Activity of FTO Necessary for Its Role in Adipogenesis

FTO is 2-oxoglutarate dependent demethylase of single stranded nucleic acids [63]. Its main substrate is likely 6-methyladenosine (m6A) in RNA [64]. In 3T3L1 preadipocytes, overexpression of full length wild type* Fto* enhanced adipogenesis whereas overexpression of catalytically inactive R96Q* Fto* did not affect adipogenesis [60]. Similarly, the effect of* Fto* knockdown on adipogenesis in 3T3-L1 cells could be rescued by reexpressing wild type* Fto*, but not by reexpressing catalytic inactive* Fto* [58]. The reduction in cellular proliferation during the mitotic clonal expansion phase of adipogenesis in* Fto*-KO MEFs could be rescued by expressing WT* Fto*, but not by catalytic inactive R313A* Fto* [38].

In porcine adipocytes, overexpression of* FTO* increased m6A levels, and knockdown of* FTO* reduced these levels. Interestingly, overexpression of the m6A methylase* METTL3* induced similar effects of* FTO* knockdown, whereas knockdown of METTL3 had no effect. Chemically increasing (via methyl donor betaine) m6A levels mimicked* FTO* knockdown, whereas chemical reduction of m6A levels (via methylation inhibitor cycloleucine) reflected the effects seen with* FTO* overexpression [61]. In another study, knockdown of* ALKBH5*, another m6A demethylase, did not affect adipogenesis. Knockdown of* METTL3* on the other hand increased lipid accumulation during adipogenesis [58]. So, m6A is likely to play a role in adipogenesis, but the effect is substrate-specific, as FTO's catalytic activity is essential for adipogenesis; however, that of ALKBH5 is unlikely to be important.

### 4.2. Mechanism via Which FTO Affects Adipogenesis

FTO was shown to influence adipogenesis at an early stage indeed, during mitotic clonal expansion, which takes place during the first 48 hours after adipogenic stimulation* in vitro* [38]. A potential mechanism via which FTO regulates adipogenesis is RUNX1T1. The SR proteins are important for splice site recognition and intron processing, and FTO was shown to regulate the splicing of SRFS2 target genes via modification of m6A levels. One of these target genes is* RUNX1T1*, which has 2 isoforms. The short isoform is proadipogenic, whereas the long isoform reduces adipogenesis. FTO knockdown decreases the expression of the short isoform [58]. RUNX1T1 has been shown to regulate C/EBP*β* activity [65]. Interestingly enough, FTO was shown to act as a transcriptional coactivator for the transcriptional regulators C/EBP*α*, C/EBP*β*, and C/EBP*δ* [66]. One study found that* FTO* deficiency did not affect the expression levels of C/EBP*β*, suggesting FTO acts via a C/EBP*β* independent route [39]. But FTO is likely to affect the activity of C/EBP*β*, rather than its expression.

## 5. FTO and Lipogenesis

Besides adipogenesis, FTO might play a role in lipogenesis. In one study, knocking down* FTO* in the human preadipocyte SGBS cells did not affect adipogenic differentiation, lipolysis, or glucose uptake but did attenuate de novo lipogenesis [67]. And in human myotubes,* FTO* overexpression caused an increase in lipogenesis and upregulation of the expression of genes involved in lipogenesis [57]. Church and colleagues reported a gross increase in adipocyte size after prolonged high fat diet feeding in mice [37], and as adipogenesis also relies upon lipogenesis to assemble lipids into triglycerides during terminal differentiation, these findings suggest that FTO may play two distinct roles in adipogenesis (during MCE at the start and in lipid filling at the culmination of the process). A recent study showed a link between lipogenesis and adipogenesis via the carbohydrate-response element-binding protein (chREBP) which was shown to regulate* PPARγ* [68]. It is conceivable that FTO may influence chREBP and it would be interesting to assess the effects on* chREBP* expression in preadipocytes and MEFs from* Fto*-KO and* Fto* overexpression mice.

## 6. FTO Deficiency and Browning of White Adipose Tissue

An obesity-associated SNP in intron 1 of* FTO* has been associated with decreased browning of white adipose tissue, which coincided with an increase in expression of* IRX3* and* IRX5* in preadipocytes [29]. Browning of WAT has also been reported in* Irx3*-KO mice [28]. Furthermore, also* Fto*-KO mice showed signs of WAT browning. Expression of* Ucp1* in gonadal and inguinal fat pads was increased in* Fto*-KO mice, and* Fto* deficient adipocytes showed increased expression of* Ucp1* and increased mitochondrial respiration [67]. Furthermore,* FTO* variants were suggested to influence adipose tissue lipolysis and metabolism [24, 46]. Given that* Fto* deficiency is associated with increased circulating noradrenaline levels [31, 34, 67], increased thermogenic capacity and lipolysis of* Fto* deficient adipocytes may result from increased adrenergic receptor activation.

## 7. FTO and Developmental Programming

Given that* Fto* deficiency results in developmental abnormalities consistent with postnatal growth retardation [31], characterized by reduced lean mass and body length, it would be appropriate to postulate that FTO may act developmentally to influence body composition. Interestingly, Dina and colleagues demonstrated that FTO SNPs are associated not only with adult obesity but also with childhood obesity [8]. In fact, the association between* FTO* SNPs and obesity peaks during early adolescence and tapers off thereafter [45, 69–71], suggesting that* FTO* SNPs may impact obesity by influencing events during the developmental period. Interestingly, one study found that the* FTO* risk allele is actually associated with a reduction in BMI under the age of 2.5 years, and with a shift in the timing of the BMI adiposity rebound, which is a developmental marker. The obesity risk alleles accelerate the developmental age, which subsequently results in an increase in BMI during later time-points [71].

A recent study in rats found that* FTO* mRNA expression in the hypothalamus was increased in offspring following maternal obesity and was associated with a predisposition to high fat diet induced obesity in adulthood, potentially due to increased food intake [72]. It remains to be elucidated whether a similar expression pattern is observed with maternal obesity in offspring adipose tissue, although maternal nutrient restriction had no impact on offspring adipose tissue mRNA levels [73]. Epigenetic processes have been shown to be highly active during the developmental period and may represent a mechanism independent of changes in the DNA sequence by which maternal diet may impact offspring obesity susceptibility. Interestingly, FTO risk allele rs8050136 has been shown to have increased methylation on a per-allele basis [74]. This could represent an exciting new mechanism by which* FTO* SNPs affect obesity and warrants further investigation. Furthermore, a very recent study observed a parent-of-origin effect of* FTO* SNPs, albeit in a very small population in Germany, suggesting that these parent-of-origin effects may modulate the association between* FTO* SNPs and obesity [75].

Recently, it was shown that adipogenesis during development and obesity are regulated by distinct signalling pathways and utilize separate preadipocyte populations (both of which are marked by birth) [62]. Adulthood adipogenesis in mice, which only occurs under HFD conditions, requires Akt2/PI3K signalling and requires smooth muscle actin (SMA) positive preadipocytes, whereas developmental adipogenesis (organogenesis) is not dependent on this pathway [62]. To this end, we have shown that* Fto* overexpression does not affect developmental adipogenesis but has profound effects on obesogenic adipogenesis [38]. Interestingly, in endometrial cancer cells, *β*-estradiol- (E2-) induced proliferation and invasion were shown to be mediated by FTO. E2 stimulated* FTO* expression via the PI3K/Akt and MAPK signalling pathways.* FTO* knockdown attenuated cancer cell growth and proliferation which was mediated via cyclin D1 regulation [76]. This is particularly intriguing as FTO has been shown to induce adipogenesis through augmenting cyclin D1 expression during the MCE phase [38]. These studies thus indicate that AKT/PI3K signalling may be a crucial part of the as yet unidentified signalling process of FTO-mediated adipogenesis; however, more direct evidence is required to confirm this hypothesis.

## 8. Conclusion

Whether or not alterations in* FTO* expression are responsible for the obesity-associated SNPs in intron 1 of* FTO* remains to be unequivocally answered. Nevertheless, FTO clearly plays an important role in adipogenesis. Given the similarities between* Fto* and* Irx3* animal models, these genes might act well in concert to regulate adipogenesis and the occurrence of browning of white adipose tissue. The discovery of FTO as a demethylase of single stranded DNA and RNA, and its target m6A, has revealed the importance of m6A in RNA and how it is crucial for important physiological processes, such as adipogenesis. Future studies will address the differences between FTO's roles in development and in adulthood, not only in adipose tissue but also in other tissues.
